# Radiological diagnosis of dialysis-associated complications

**DOI:** 10.1007/s13244-014-0350-4

**Published:** 2014-08-06

**Authors:** Shahin Zandieh, Dina Muin, Reinhard Bernt, Petra Krenn-List, Siroos Mirzaei, Joerg Haller

**Affiliations:** 1 Institute of Radiology and Nuclear Medicine, Hanusch Hospital, Teaching Hospital of Medical University of Vienna, Vienna, EU Austria; 2 Department of Internal Medicine, Division of Nephrology and Hemodialysis, Hanusch Hospital, Teaching Hospital of Medical University of Vienna, Vienna, EU Austria; 3 Institute of Nuclear Medicine with PET-Center, Wilhelminenspital, Teaching Hospital of Medical University of Vienna, Vienna, EU Austria

**Keywords:** Dialysis, Complications, Infections, Shunt stenosis, Catheter dysfunction, Cardiovascular diseases

## Abstract

In daily clinical practice, the radiologist in the context of diagnosis often faces dialysis-associated complications. The complications are numerous and range from infections, catheter dysfunctions, haematomas, cardiovascular diseases, digital ischaemia, and pseudoaneurysms to shunt stenosis. In this pictorial essay, we take a close look at the imaging diagnostics of the most common complications in dialysis patients.

*Teaching Points*

• *The occurrence of venous stenosis in haemodialysis patients is up to 41 %.*

• *Catheters usually have a fibrin sheath that can be rinsed but not aspirated.*

• *The steal phenomenon occurs in 75–90 % of patients with a shunt system.*

• *Arterial pseudoaneurysms can cause a number of complications.*

## Introduction

The number of patients requiring dialysis treatment is continuously increasing, correlating with the increased incidence of heart diseases and diabetes [1]. Haemodialysis, peritoneal dialysis (PD), and kidney transplantation are available as renal replacement therapies. Dialysis treatment results in both increased life expectancy and better quality of life.

As with any treatment, there are method-specific complications. Complications of haemodialysis are mostly problems related to the vascular access, as seen with the onset of stenosis, thrombosis, and infection of the shunt system. Whereas thrombotic occlusion and infections or circulatory problems are the primary causes in regard to central venous catheters, the complications of peritoneal dialysis are mostly catheter-related. Infections of the catheter exit point and catheter tunnel, peritonitis, as well as insufficient ultrafiltration and insufficient clearance in the course of time are the most essential problems. Very different information on this subject can be found in the literature reports, where no age-dependent difference in the incidence of peritonitis but advantages and disadvantages for patients above 65 years of age [2, 3] are reported. Due to the increasing number of patients who are elderly and have multi-morbid conditions, the aforementioned complications are occurring more frequently. This results in the necessity of constant medical care for dialysis patients. In daily clinical practice, in the context of diagnosis, the radiologist faces a number of typical complications. In this study, we would like to take a close look at the imaging diagnostics of the most common complications in dialysis patients.

Effective haemodialysis requires flow rates of more than 300–400 ml/min. As a temporary means of vascular access, usually only used in emergencies, especially due to acute renal failure or a fistula thrombosis, a Shaldon catheter, named after its developer, is mostly used today. The catheter allows haemodialysis via two separate lumina. The catheter is primarily positioned in the superior vena cava [vena cava superior (VCS)] or occasionally in the femoral vein [4].

For permanent treatment, the Demers-Katheter®, named after Dr. Demers and developed in Darmstadt, is most suitable. This catheter is fitted with subcutaneous tunneling and an ingrowth cuff. Thus, the incidence of infections should be reduced. This catheter is placed into the VCS, like the Shaldon catheter. The tip of the catheter should thus be in the right atrium to ensure adequate flow [5].

Dialysis fistula or dialysis shunt refers to a permanent, surgically created connection between a vein and an artery. This type of catheter grew in importance with the successful installation of the Cimino-Brescia fistula [6]. If no usable vessels can be located in the patient, vessels made of plastic can be used. These consist of simple, small-volume tubes with well-tolerated material [polytetrafluoroethylene (PTFE) such as Gore-Tex®].

In contrast to haemodialysis, PD uses the peritoneum as a natural filter membrane, so the patient is dialysed. The use of PD has increased significantly in recent years. It operates on the same principle as haemodialysis, but it bears a relatively high risk of infection and requires good compliance.

## Complications of the catheter system

### Arterial failed puncture

Risk factors for the acute occurrence of an arterial failed puncture during the insertion of a dialysis catheter are prior irradiation of the implanted region or postoperative changes at the insertion site [7]. In addition, patient obesity and lack of expertise by the interventional radiologist play important roles [7]. The error rate of failed arterial puncture without the aid of ultrasound is 8.4 % [8]. The incidence of error is higher for the internal jugular vein (VJI) than for the subclavian vein (VS) [9]. Up to 30 % of failed arterial punctures can become symptomatic and cause complications, as seen with the onset of haematoma, haemothorax, and neurological deficits [9]. The optimal treatment management ranges from haemostasis by compression to vascular surgery or an endovascular intervention [10]. Since it is generally difficult to compress the subclavian artery manually, aside from a vascular surgical intervention, arterial occlusion by a suture, clip, or collagen-based closure system is possible [10].

### Haematoma

**Figs. 1 and 2:**
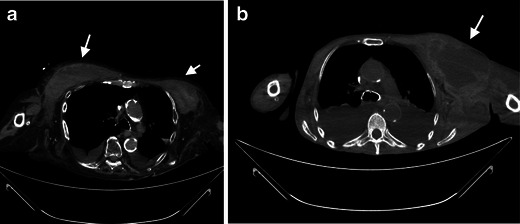
A 43-year-old female patient with a newly placed central venous catheter. Non-contrast CT of the upper thorax with axial reconstruction illustrates unsuccessful catheter implantation with a space-occupying lesion in the left and right chest wall (*white arrows*), which is partly solid, as seen with haematoma

After implantation of the dialysis catheter, minor bleeding from the puncture point occasionally occurs, which can be treated, usually without much effort (Figs. 1 and 2).

The incidence of haematoma varies from 0.5 to 6.1 % [11–13]. Causes may include multiple puncture attempts, coagulopathies, or an incorrect technique. Occasionally, drainage or surgical removal is required for a larger haematoma.

### Incorrect catheter position

**Fig. 3:**
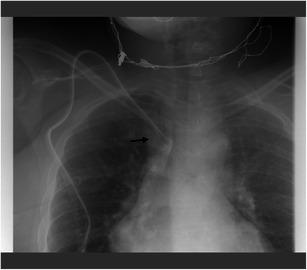
The chest x-ray (anterior-posterior view) of a 50-year-old female shows the incorrect position of the catheter. The catheter tip is displayed proximally in the area of the right innominate vein (*black arrow*)

**Fig. 4:**
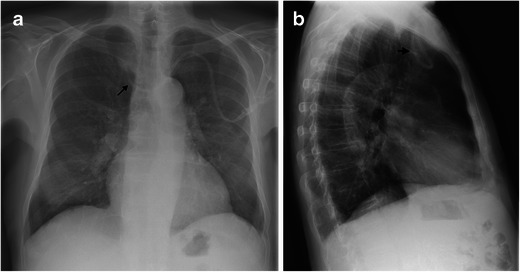
**a**, **b**. On the chest x-ray (posterior-anterior and lateral views) of this 87-year-old male patient with a new central catheter set to the left, the incorrect position becomes visible. The catheter tip is turned upward in the right innominate vein (*black arrow*)

**Fig. 5:**
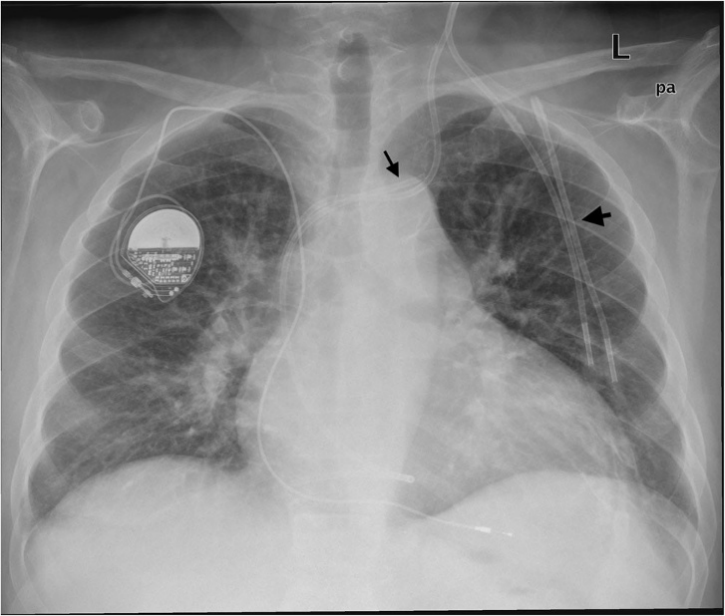
A 67-year-old male patient with a broken central catheter on the left on chest x-ray (PA view). The distal part of the broken central catheter is dislocated in the area of the right ventricle (*black arrows*). The second central catheter on the left is in the regular position

**Fig. 6:**
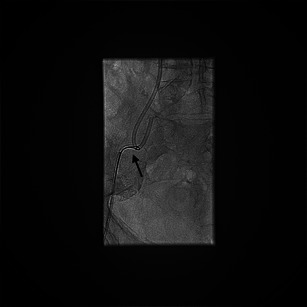
Angiographic intervention for correcting the incorrect position of a central catheter in a 74-year-old male patient. After local anaesthesia, the femoral vein is punctured in the right groin. By means of a guide wire and catheter probing of the left innominate vein, the dislocated catheter is grasped with a sling and removed (*black arrow*)

**Fig. 7:**
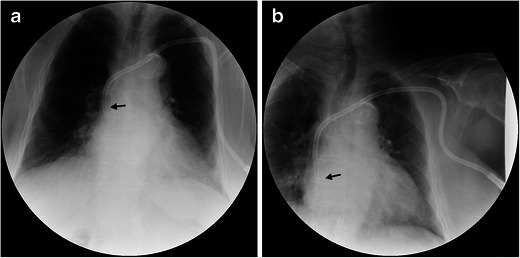
**a**, **b**. Fluoroscopy of this 70-year-old male patient shows a floating dialysis catheter. The catheter tip is displayed lying on the border of the atrium ventricle. When seated, the catheter tip migrates into the innominate vein (*black arrow*)

Dysfunction is usually caused by incorrect position of the central catheter. Thus, no adequate blood flow can be generated. The usual procedure attempted is to reposition the catheter. If this is not possible, the implantation of a new catheter is necessary. The localisation is usually determined based on an x-ray (Figs. 3, 4a, b, 5, 6 and 7a, b).

### Pneumothorax

The incidence of pneumothorax is stated to be 0–3.3 % in the literature [10, 14]. Pneumothorax is a dreaded complication that can cause a life-threatening situation. Prompt and adequate treatment is required. Drain implantation may occasionally be required. Pneumothorax occurs more often when inserting the catheter via the VS than via the VJI [7].

### Air embolism

Air embolism occurs rarely; its incidence is stated to be up to 0.8 % in the literature [14, 15]. It can be observed directly under fluoroscopy, by the acoustic, audible escape of air from the vascular sheath, and by a sudden decrease in saturation. Symptomatic air embolism can be treated by the Trendelenburg position, increasing of the central pressure by volume addition, the Valsalva manoeuvre, and the administration of 100 % oxygen. The mortality rate is 23–50 % [16, 17].

### Nerve injuries

Nerve injury is a rare complication with an incidence of 1.6 % [18]. It may result in injuries to the brachial plexus, sympathetic trunk, and recurrent laryngeal nerve with the result of vocal cord paralysis [19–23]. The reasons for this are mostly direct trauma caused by the puncture needle, pneuropraxia by compression of the nerves because of haematoma, and fibrosis [22–24]. Function recovery can take up to 12 months [22].

### Cardiac arrhythmia

In very rare cases, inserting the catheter via a guide wire can lead to cardiac arrhythmias, which are often asymptomatic. The risk of heart block or cardiac arrest is low, however [9]. The incidence of atrial fibrillation ranges from 0.1 to 0.9 %. It is usually caused by the irritation of the right atrial wall by the catheter tip. Persistent arrhythmia is treated pharmacologically or by electrocardioversion. Catheter removal is imperative.

### Catheter-associated stenosis and thrombosis

Venous catheter-associated stenosis is a common complication. The occurrence of venous stenosis in haemodialysis patients is stated to be up to 41 % in the literature [25]. Mechanisms that can lead to the induction of venous stenosis are endothelial damage during the implantation process, damage caused by movement of the catheter tip, flow changes, and inflammatory reactions of the vessel wall. Isolated swelling of the face or an extremity is a clinical indicator of complete central venous stenosis [10]. Frequently, dilated neck veins are visible as a sign of the formation of collateral circulation. Phlebography is the gold standard for the diagnosis of stenosis. Primarily, the therapy of venous stenosis should occur symptomatically. Venous collaterals generally form rapidly, providing venous effluent and alleviating the symptoms. If the affected vein is needed for further haemodialysis and the symptoms do not weaken with the therapy, percutaneous transluminal angioplasty (PTA) is indicated for these patients to allow reimplantation [26]. If a single angioplasty does not promise success, then stent implantation should be considered. Another risk factor for the development of venous stenosis is a twisted course of the dialysis catheter in the body. Thus, they are more common with catheters implanted via the VS than via the VJI because the straight course of the VJI has a low risk for the development of stenosis [27].

**Fig. 8:**
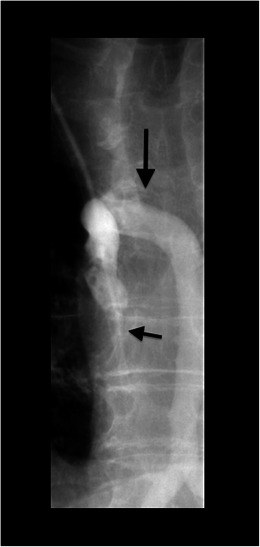
Fluoroscopy of a 78-year-old male patient after contrast media application. The contrast media builds up in the superior vena cava around the catheter, indicating a larger thrombus formation that could occlude the superior vena cava (*black arrow*). The contrast media outflow from the arterial leg occurs distally via the hemiazygos vein (*black arrow*)

**Fig. 9:**
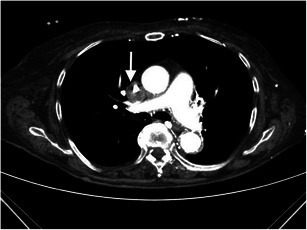
An 84-year-old female patient with a central venous catheter. Contrast CT of the upper thorax with axial reconstruction. The most striking finding is shown on both the shoulder girdle and in the thoracic wall in the area of the internal mammary group, where there are extensive mediastinal collateral veins with large calibre and a strongly contrasted azygos vein. The internal jugular veins show a slight difference, with a larger calibre right versus left internal jugular vein in the neck portion; however, free contrast is seen on both sides. Finally, a contrast media segment appears in the area of the vena bracheocephalica after the confluence of the innominate vein on the superior vena cava close to the wall, where the remainder of the vessel around the Permcath, which has been inserted from the right, is more distended and does not show contrast (*white arrow*). This primarily corresponds to a longer vena cava superior thrombosis segment around the catheter

The activation of blood coagulation occurs through dialysis catheters, which represent an intravascular foreign object. The incidence of catheter-associated thrombosis depends on numerous risk factors. Catheter material, catheter diameter, and a complication-rich catheter implantation with consecutive endothelial damage predispose patients to the formation of venous thrombosis. The incidence of catheter-associated pulmonary embolism is 15–25 % [28]. Primary thromboprophylaxis has a dubious value in oncological patients and no significant benefit [29]. Occasionally, catheter-associated thrombosis may cause pressure-sensitive swelling of the affected extremity. Sonography or a phlebography is required for diagnostics. The catheter should be removed in the case of symptomatic thrombosis. If the catheter tip is free of thrombotic deposits, the catheter can remain in place. The thrombosis of the catheter-bearing vein should be treated. Therapy with anticoagulation is essential to prevent progression of the deposits. Oral anticoagulation for up to 6 months with an optimal level of 2 to 3 of the international normalised ratio (INR) is recommended. In severe cases, thrombolytic therapy should be initiated. A fibrinolytic applied directly into the thrombus has an advantage over systemic or regional thrombolysis because less fibrinolytic agent is needed and thus fewer side effects occur [30]. Mechanical thrombectomy should occur either surgically or by means of percutaneous aspiration thrombectomy or fragmentation catheters (Figs. 8 and 9).

**Fig. 10:**
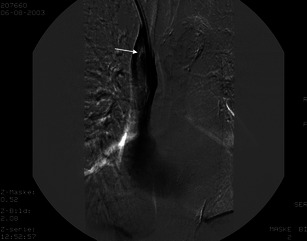
The fluoroscopy of a 65-year-old male patient after contrast media application shows lamellar contrast defects around the tip of the dialysis catheter, as seen with the onset of a fibrin sheath (*white arrow*)

In dialysis catheters, a fibrin coating can form that wraps around the catheter. A definite cause for the formation of the fibrin sheath is unclear. The fibrin sheath can extend to the catheter end and close it. The intravascular portion of the haemodialysis catheter should be suspected to have a fibrin sheath when it can be rinsed but not aspirated. After the catheter implantation, a fibrin sheath can form after only 24 h, with an incidence of 13–57 % [31]. The treatment measures range from fibrinolysis or stripping of the fibrin sheath to perforation of the fibrin sheath by means of a guide wire. Fibrinolysis is preferable to the stripping of the fibrin sheath because it is not invasive; moreover, it is less expensive and can be administered in a hospital room. This treatment method is often preferred by patients, and it has a lower rate of complications. The success rate of urokinase is reported to be 76–97 %, without adverse complications [32–35]. Mechanical removal (stripping) can be offered to patients as an alternative where fibrinolysis is unsuccessful. Here, the catheter end is grasped through a femoral-inserted sling and is freed by pulling of the fibrin material. The disadvantage here is the high cost of the grasping sling. With an open end in the catheter, catheter function can be restored with perforation of the fibrin sheath by means of a guide wire. If the above measures are not successful, a catheter exchange is imperative for adequate haemodialysis, whereby the existing fibrin layers in the vessel must be severed using an angioplasty balloon [36] (Fig. 10).

### Infection

One of the most important and common complications is infection. It leads to increased mortality and morbidity and high costs [37, 38]. *Staphylococcus* aureus and candida have a higher affinity for polyvinylchloride catheters than for Teflon catheters. Long-term catheters do not come out of the skin exactly at the puncture site; they are partially guided under the skin before they emerge from the skin surface.

During implantation, germ transmission can occur because of non-sterile conditions; cutaneous germs can penetrate even at the exit site. At the exit site and in a possibly existing tunnel, an inflammatory reaction can occur. From there, bacteria may be transported into the bloodstream. Infections that occur within 10 days after implantation are usually caused by skin flora and subsequently by intraluminal catheter colonisation [39–41].

The rate of infection increases exponentially with the time of use of the catheter [42]. Non-tunneled catheters are associated with a very high infection rate in the 2nd to the 4th week. In VJI catheters, the incidence of bacteremia after 3 weeks is 5.4 % and increases to 10.3 % within 4 weeks after implantation [43]. Strict observation of the hygienic measures is an important approach in the implantation of a dialysis catheter. Antibiotic therapy should always be administered together with the removal of the dialysis catheter.

### Complications of a peritoneal dialysis catheter

#### Catheter dysfunction

**Fig. 11:**
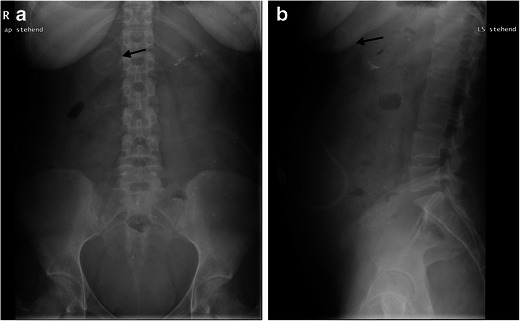
**a**, **b**. Abdominal x-ray (AP and lateral views) of this 27-year-old female patient demonstrates incorrect positioning of a peritoneal dialysis catheter in the region of the right upper abdomen (*black arrow*)

Dysfunction is usually caused by the incorrect position of the central catheter. Thus, no adequate blood flow can be generated. In such cases, it is typical to attempt to reposition the catheter. If this is not possible, the implantation of a new catheter is necessary. Its tip should be in the pelvis to accomplish adequate dialysis. The volume of the dialysate drain from the catheter tip is significantly lower in the middle abdomen than in the lower abdomen. The location is usually determined by means of an x-ray (Fig. 11a, b).

#### Haematoma

After implantation of the PD, minor bleeding from the puncture site occasionally occurs, which can usually be treated without much effort.

#### Dialysate leaks

**Fig. 12:**
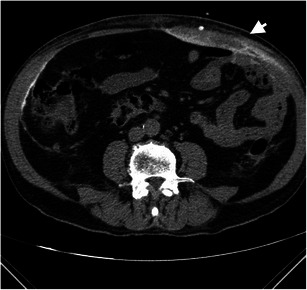
Non-contrast CT of the abdomen with axial reconstruction in an 80-year-old male patient. Accumulation of contrast media administered via a peritoneal catheter is seen in the area of the straight and oblique abdominal muscles on the left side in close relationship to the entry of the catheter through the abdominal wall (*white arrow*) and on the right side along the oblique abdominal muscles, here probably located subfascially. The change is caused by a leak

A dialysate leak as a complication occurs in more than 5 % of cases of dialysis patients and is commonly of no clinical significance [44]. It can occur in an early form within 30 days, whereby the etiology is usually catheter related, or in a late form after 30 days, often because of a mechanical or surgical rupture in the peritoneal membrane. With increased intra-abdominal pressure because of the dialysate, the likelihood of occurrence of a leak from the peritoneal cavity increases. Other mechanisms that increase intra-abdominal pressure, such as coughing or obesity, may also be responsible for leaks [44, 45] (Fig. 12).

The access point of the catheter represents an obvious weakness of the abdominal wall. Other potential locations for a leak are weak points in the abdominal wall from surgical defects that occurred, pleuro-peritoneal connections in the thoracic cavity associated with pleural effusion, and persistent processus vaginalis [46]. Every now and then, there may be a retroperitoneal leak, which is rarely recognised clinically. However, it is of great importance because it can be the cause of acute dialysis insufficiency, which may impair a dialysis treatment, and in some cases, it even requires surgical intervention [47].

#### Hydrothorax

A dialysate leak in the pleural cavity can result in a hydrothorax, a rare but potentially life-threatening complication that can occur shortly after the initiation of peritoneal dialysis or several years afterward. It occurs most frequently on the right side [48]. Dyspnoea is the earliest clinical manifestation of a pleuro-diaphragmatic leak. A leak can result from diaphragmatic defects or lymphatic transport via the diaphragm. The diagnosis of a pleuroperitoneal leak is confirmed by pleural fluid analysis with a high glucose concentration in the transudate and a normal glucose concentration in the blood [49]. A pleural effusion is visible when taking x-rays of the lung. An interruption of the peritoneal dialysis can result in a reduction of the hydrothorax after 2 to 6 weeks. Surgical intervention or pleurodesis is indicated for a recurrent dialysate leak. Pleurodesis is used more often for a lymphatic leak than for a diaphragmatic leak [48].

#### Infections

**Fig. 13:**
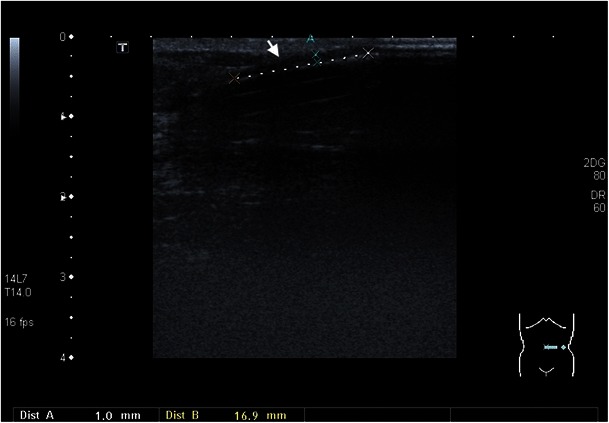
In a 51-year-old male patient with elevated inflammatory laboratory parameters, ultrasound examination shows a hypoechoic structure along the dialysis catheter in the subcutis, which corresponds to inflammatory changes (*white arrow*)

**Fig. 14:**
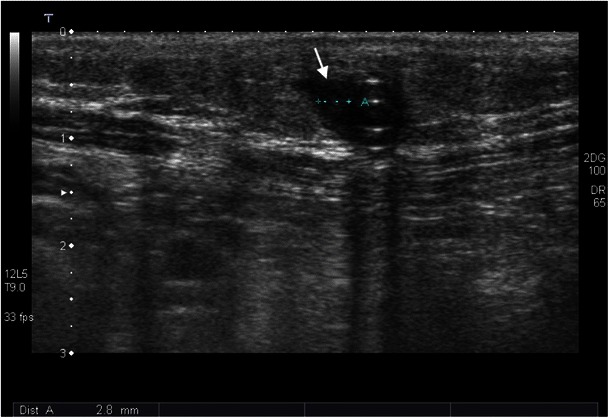
Ultrasound examination demonstrates a hypoechoic formation around the dialysis catheter in a 23-year-old female patient with tunnel infection (*white arrow*)

**Fig. 15:**
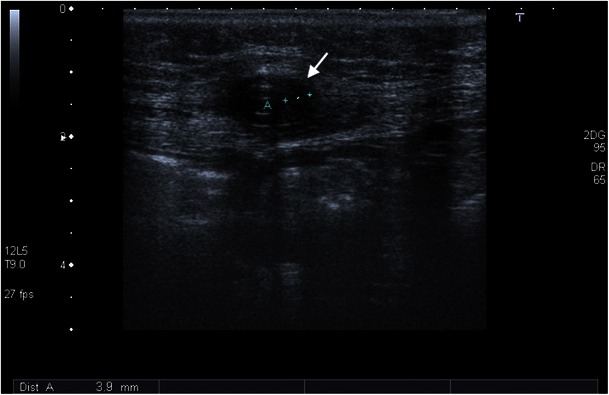
Allergic reaction to the catheter material, which led to eosinophilic peritonitis. A hypoechoic formation around the catheter is recognisable on the ultrasound images of this 71-year-old female patient (*white arrow*)

**Fig. 16:**
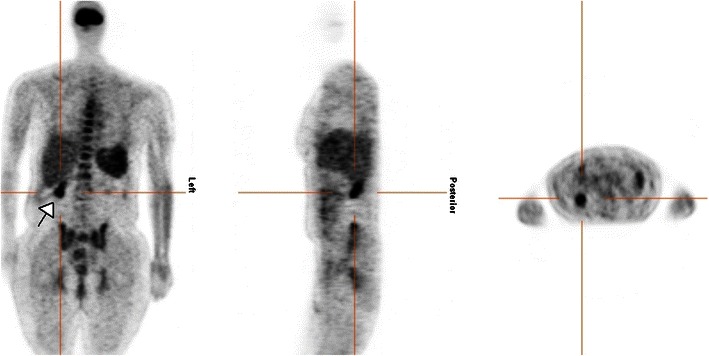
18F-FDG PET examination with axial, sagittal, and coronary reconstruction depicts focal uptake in the right upper abdomen in a 65-year-old female haemodialysis patient in the context of a peritoneal infection (*white arrow*)

In peritoneal dialysis, bacterial infections often result in peritonitis, which usually manifests in fever and abdominal pain. The standard therapy for bacterial infections is antibiotic therapy without interruption of the dialysis. However, this does not always lead to the desired success. In therapy-resistant cases, the catheter should be removed. There is a possibility that bacteria may grow in the interior of the catheter, especially when the work is done in an unhygienic manner; blood runs back into the catheter and is not rinsed out immediately. As a result, tiny clots often form on the catheter wall, providing a suitable breeding ground for bacteria. They can enter the bloodstream and cause fever during the next infusion. Occasionally, a general infection (sepsis) may arise with complications such as spondylodiscitis or endocarditis. As a rule, in such a case, the catheter is removed; however, antibiotic therapies may also be attempted. About 39 % of catheter removals are because of an infection at the exit site or a tunnel infection that does not respond to antibiotics [50]. Clinically, it is important to recognise whether a catheter infection exists at the exit site, since it has a greater risk of not responding to antibiotics and more often results in catheter removal [51, 52]. Infections at the exit site and tunnel infections are characterised by erythaema and pressure pain. Especially with PD, ultrasound is very well suited for the detection of latent infections [51, 52]. The application of ultrasound is enormously important during follow-up, because in case of persistence of fluid collection over a period of more than 2 weeks of antibiotic shielding, a catheter change is recommended. Otherwise, there is a risk of peritonitis (Figs. 13, 14, 15, and 16).

#### Hernias

**Fig. 17:**
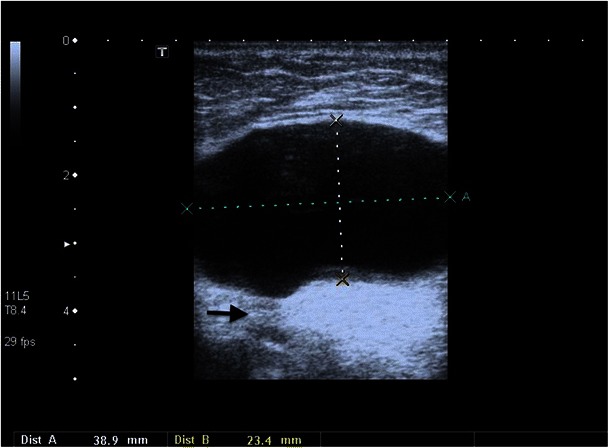
A hernial opening in the abdominal wall with a sharply defined hernia sac of about 4 cm is recognisable on the ultrasound images of this 72-year-old female patient (*black arrow*)

**Fig. 18:**
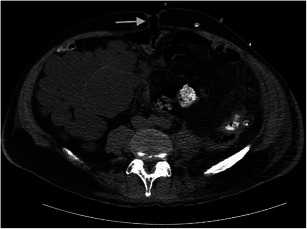
Contrast-enhanced CT scan with axial reconstruction shows a hernial opening with a hernial sac median in the abdominal wall of this 69-year-old male patient (*white arrow*)

Hernias are a frequent complication because of an increase in intra-abdominal pressure and peritoneal defect generated by the catheter implantation, which occur in up to 25 % of the cases of dialysis patients, mostly umbilical next to the peritoneal dialysis catheter and in the inguinal channel [53]. They can lead to a dialysate leak and incarcerations and strangulation of bowel loops, which usually require surgical intervention (Figs. 17 and 18).

#### Subcapsular hepatic steatosis

Subcapsular hepatic steatosis is a rare form of accumulation of fat within the liver, first described in 1989 by Wanless et al. [54]. According to the literature, it occurs in about 18 % of peritoneal dialysis patients [55]. It is caused by the contact of subcapsular hepatocytes with insulin of the dialysate injected into the peritoneal cavity. On ultrasound, it can be identified as echogenic nodules or scar tissue or as hypodense subcapsular areas on CT, generating a signal drop on the opposed-phase weighted MRT images. Termination of dialysis can lead to a regression [55]. Normally, no malfunction of the liver is caused, and the disease is of no clinical significance. However, it is appropriate to recognise these areas properly to prevent further unnecessary examinations.

#### Complications of the shunt system

##### Digital ischaemia

**Fig. 19:**
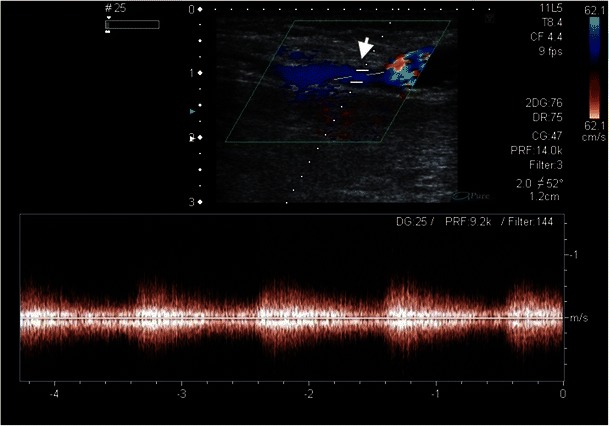
Distal to the arteriovenous fistula, retrograde flow is recognised on duplex sonography in the radial artery, which indicates the steal phenomenon in this 58-year-old male patient (*white arrow*)

The steal phenomenon occurs in 75–90 % of patients with a shunt system [56–58]. Usually, the steal phenomenon is clinically silent, and the patient remains asymptomatic. The steal phenomenon becomes steal syndrome when compensatory mechanisms to maintain peripheral arterial perfusion fail. Risk factors for a steal syndrome are female gender, age over 60 years, and diabetes mellitus [59]. Steal syndrome is characterised by pain at rest, pain during haemodialysis sessions, ulcer formation, and mostly acral necrosis. The most common form of steal syndrome is a fistula with a high flow volume, the so-called high-flow fistula. The retrograde flow drains so much blood from the extremity peripheral of the fistula that ischaemia results. The cause is usually venous resistance that is too low (Fig. 19).

##### Cardiovascular diseases

**Fig. 20:**
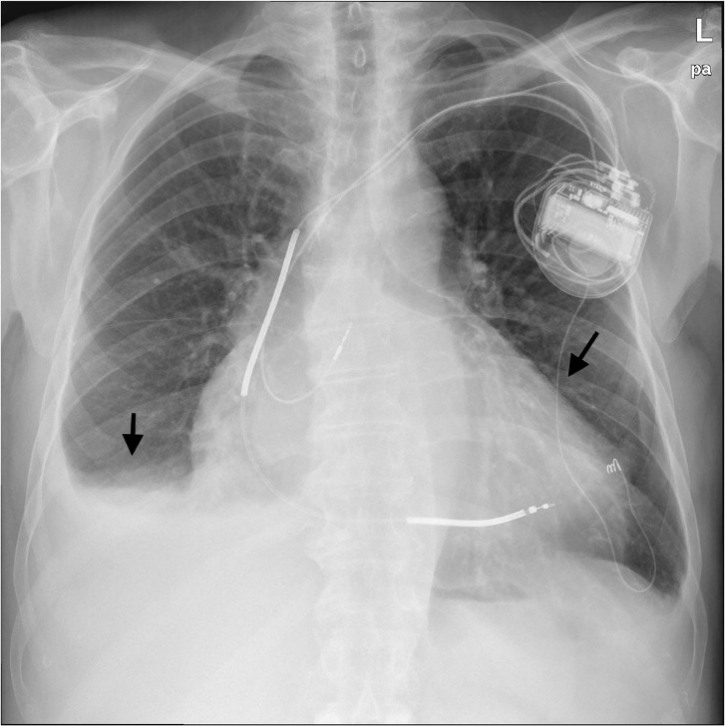
Chest x-ray depicts left ventricular hypertrophy in a 79-year-old male patient with a dialysis shunt and increased cardiac output (*black arrow*)

Another group of possible complications during dialysis affects the heart. Cardiovascular diseases are common causes of death among dialysis patients [60]. During dialysis, pericarditis with the accumulation of fluid in the pericardium can occur. This leads to a compression of the heart, which then can no longer move optimally [61, 62]. In such cases the pericardial effusion should be punctured. Cardiac arrhythmias and left ventricular hypertrophy often arise in dialysis [63] (Fig. 20).

##### Pseudoaneurysms

**Fig. 21:**
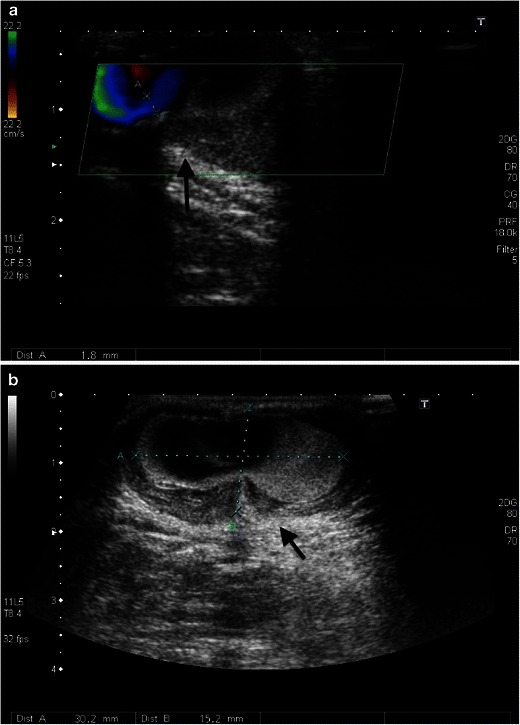
**a**, **b**. An aneurysma spurium is shown on duplex sonography in an area of palpable resistance laterally on the middle forearm in this 69-year-old female patient (*black arrow*). The aneurysm neck is 2 mm wide

**Fig. 22:**
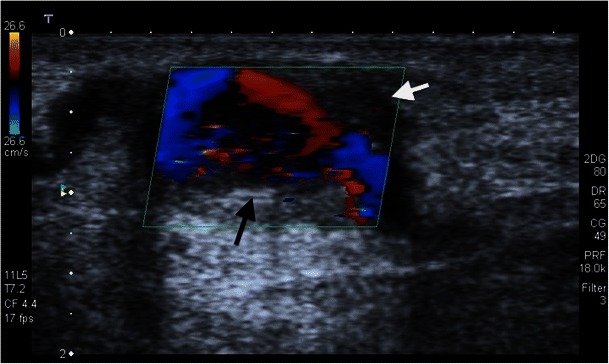
In an area of palpable swelling on the left forearm, the radial artery shows an aneurysmatic enlargement with a length of approximately 2 cm and a width of about 1 cm on duplex sonography in this 53-year-old male patient (black arrow). It then becomes regularly narrower. Additionally, thrombotic deposits are shown in this region (*white arrow*)

**Fig. 23:**
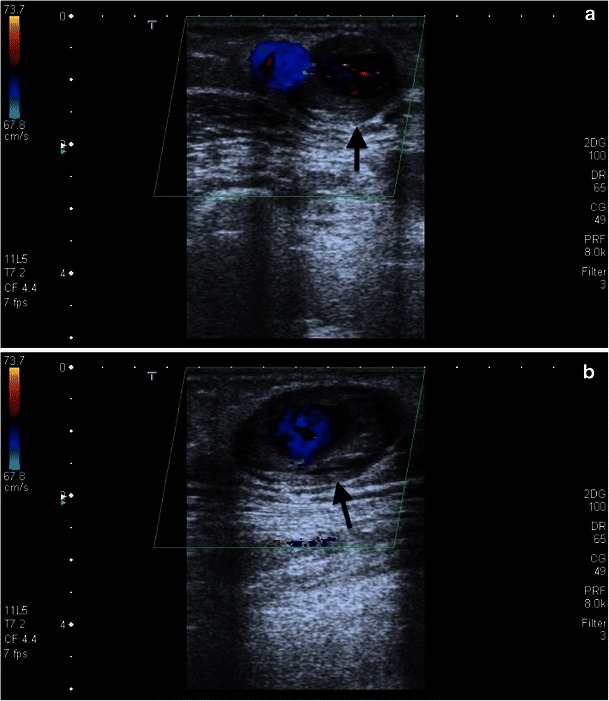
**a**, **b**. The AV fistula (Gore-Tex sling) is open in this duplex sonography image from a 57-year-old female patient. In the distal third of the venous portion, located between the two puncture sites, a partially thrombosed aneurysm spurium can be identified (*black arrow*)

Multiple punctures of the vessel wall can result in a circumscribed area in aneurysms or pseudoaneurysms. They can arise from venous segments or the original artery. Venous pseudoaneurysms have a low complication rate, but arterial pseudoaneurysms can cause a number of complications, such as ectasia, rupture, arterial thrombosis, and steal syndrome. Percutaneous ultrasound-aided thrombin injection is usually more effective than ultrasonic-assisted compression (Figs. 21a, b, 22 and 23a, b).

##### Shunt stenoses

Shunt stenoses are bottlenecks within a blood vessel. A stenosis may be congenital or acquired. With acquired stenoses, a distinction is made between post-traumatic stenoses, such as the so-called induced stenoses (“kinking”), and surgically created stenoses (“banding”) [64].

Post-traumatic lesions are caused by intraoperative trauma because of dissection and separation with destruction of the vasa vasorum or by lesions in the vein wall or in a venous valve caused by punctures. The induced stenoses include (sub)intimal hyperplasia due to the increased shear stress of turbulent blood flow in the shunt vein with mechanical strain on the endothelial layer and/or the underlying smooth vascular muscle layer. Consequently, it may lead to the migration of myofibroblasts and vascular smooth muscle cells from the media into the intima. They can proliferate and produce extracellular matrix [65].

**Fig. 24:**
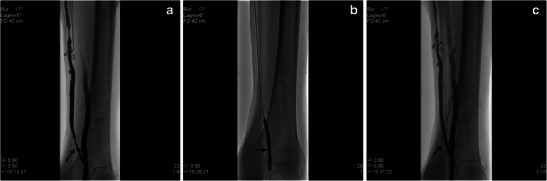
**a**, **b**, **c**. Angiography of the venous shunt in the left thigh in this 70-year-old male patient shows a proximal short segment of bottleneck just distal to the anastomosis in the distal left thigh (*black arrow*). Venous access occurs via the common femoral vein, whereby after passage of the intact vena saphena flaps, a 2-cm flow-limiting high-grade anastomosis stenosis on the transition of the POP1/POP2 segment is shown retrogradely. A balloon dilatation (PTA) could be performed without complications for significantly improved shunt function (*black arrow*)

Stenoses can be partially treated by appropriate puncture techniques. If a stenosis in the area of the fistula or the interponat is suspected, gaining dialysis access has absolute priority. The surgery should be done in such a way that at least a part of the access can be punctured immediately so the implantation of a temporary dialysis catheter can be avoided. Stenoses in the area of the arterial anastomosis, so-called type I stenoses, can be treated interventionally. In addition to percutaneous transluminal angioplasty (PTA), particularly for scarred stenoses, a “cutting balloon” technique can be applied to treat the stenosis and to improve the arterial inflow. Type II stenoses, which occur in the area of the fistula vein at the puncture site, should be treated interventionally. Thus, after PTA, the puncture area can be used again immediately for dialysis. Recurrent stenoses are an indication for surgical intervention by means of a (venous) patch or (prosthetic) interponate. Stenoses of the fistula veins at their confluence into the deep venous system, type III stenoses, occasionally appear in brachiocephalic fistulas and after basilica transposition. They can be treated using PTA but have a high risk of recurrence. Here, it is possible to perform a stent implantation in the same session to preserve the dialysis access (Fig. 24a, b, c).

## Discussion

### Discussion

In recent years, there has been a reorientation away from surgery and toward interventional radiology for the insertion of dialysis catheters. Fluoroscopy improves the success rate of dialysis catheter insertion and reduces complications. Additionally, interventional radiology plays an important role in the diagnosis and management of complications of the dialysis catheter system [66].

PD is a treatment option for patients with end-stage renal failure and has been used with increasing frequency in recent years because it allows for better quality of life because of superior patient mobility and independence and because of the simplicity of its use, along with clinical advantages such as lower mortality and the maintenance of residual renal function. PD is a common alternative to haemodialysis for patients with end-stage renal disease. Infection is a common complication of PD, but this can usually be resolved with intraperitoneal administration of antibiotics without interruption of dialysis. Numerous other complications related to this method of dialysis have been described, including catheter dysfunction, haematoma, dialysate leak, and hydrothorax. Complications of PD may cause a temporary or permanent loss in dialysis functionality, with resultant impairment of the patient’s condition. The radiologist plays an important role in detecting complications at an early stage to prevent their progression [53].

Duplex sonography is a useful diagnostic method in addition to angiography because of its ability to provide a guide for radiological intervention and to be noninvasive. It provides physiologic and anatomic information for evaluating the therapeutic success of an intervention. Sonographic findings such as arterial steal syndrome are much better demonstrated sonographically than angiographically, and sonography is the method of choice when this diagnosis is suspected. It is also a useful method if graft infection is suspected. A large number of patients often have mild or transient impaired function of access fistulas. In such cases, noninvasive duplex sonographic assessment is often helpful to avoid an unnecessary invasive intervention. When duplex sonography shows no significant haemodynamic impairment, the patient does not need to undergo angiographic intervention. This may help to decrease the rate of angiographic intervention. In addition, conventional duplex and color Doppler sonography can specifically and noninvasively demonstrate and help to characterise many complications of vascular access for haemodialysis, including thrombosis, stenosis, arterial steal syndrome, and pseudoaneurysm formation [56–59].

## Conclusion

The exact imaging diagnostics of a dialysis-associated complication plays an important role in the clinical control and progression. For this reason, the radiologist should be familiar with frequent complications encountered with the respective haemodialysis system to avoid unnecessary delays in diagnosis and therapy.
